# Reflectance Spectral Images of the Retina as a Biosensor of Alzheimer's Disease

**DOI:** 10.1002/alz70856_106395

**Published:** 2026-01-08

**Authors:** Zita Salajkova

**Affiliations:** ^1^ Fondazione Istituto Italiano di Tecnologia, Roma, RM, Italy

## Abstract

**Background:**

Alzheimer's disease (AD) is a progressive neurodegenerative disorder, with current diagnostic methods being limited by invasiveness and accessibility constraints. Retinal imaging has emerged as a promising non‐invasive approach for detecting AD‐related biomarkers, given the retina's embryonic and pathological similarities to the brain. This study explores reflectance spectral imaging of the retina as a novel biosensor for early AD detection.

**Method:**

A custom‐built multispectral imaging module, compatible with commercial fundus cameras, was developed to capture retinal reflectance images in blue (<520 nm), green (520–580 nm), and red (>600 nm) spectral regions. A case‐control study included participants diagnosed with AD and age‐matched healthy subjects. Spectral intensity ratios were extracted from the images and analyzed using machine learning models, particularly XGBoost, to classify AD and healthy subjects. SHapley Additive exPlanations (SHAP) analysis was applied to determine the most predictive spectral features.

**Result:**

Significant differences in spectral intensity ratios between AD and healthy subjects were observed (*p* < 0.001). The XGBoost model incorporating spectral intensity ratios achieved high classification performance, with an accuracy of 82%, sensitivity of 87%, and an area under the ROC curve (AUC) of 0.89. SHAP analysis identified the blue spectral intensity as the most relevant predictor, suggesting its potential role in detecting AD‐related retinal alterations.

**Conclusion:**

Retinal spectral imaging offers a non‐invasive, accessible, and cost‐effective method for early AD screening. The integration of this approach with existing ophthalmic equipment presents a viable strategy for large‐scale population screening and monitoring of disease progression. Further longitudinal studies are warranted to validate the predictive value of retinal biomarkers in preclinical AD stages.

